# How to design, implement and evaluate organizational interventions for maximum impact: the Sigtuna Principles

**DOI:** 10.1080/1359432X.2020.1803960

**Published:** 2020-08-26

**Authors:** Ulrica von Thiele Schwarz, Karina Nielsen, Kasper Edwards, Henna Hasson, Christine Ipsen, Carl Savage, Johan Simonsen Abildgaard, Anne Richter, Caroline Lornudd, Pamela Mazzocato, Julie E. Reed

**Affiliations:** a School of Health, Care and Social Welfare, Mälardalen University, Västerås, Sweden; b Medical Management Centre, LIME, Karolinska Institutet, Stockholm, Sweden; c Institute of Work Psychology (IWP), University of Sheffield, Sheffield, UK; d Department of Management Engineering, Technical University of Denmark, Lyngby, Denmark; e Unit for Implementation and Evaluation, Center for Epidemiology and Community Medicine, Stockholm, Sweden; f National Research Center for the Working Enviroment, Copenhagen, Denmark; g Institute of Environmental Medicine (IMM), Karolinska Institutet, Stockholm, Sweden; h NIHR CLAHRC for Northwest London, Chelsea and Westminster Hospital, London, UK; i School of Health and Welfare, Halmstad University, Halmstad, Sweden

**Keywords:** Academy-practice partnership, occupational health interventions, participation, recommendations, workplace-based interventions

## Abstract

Research on organizational interventions needs to meet the objectives of both researchers and participating organizations. This duality means that real-world impact has to be considered throughout the research process, simultaneously addressing both scientific rigour and practical relevance. This discussion paper aims to offer a set of principles, grounded in knowledge from various disciplines that can guide researchers in designing, implementing, and evaluating organizational interventions. Inspired by Mode 2 knowledge production, the principles were developed through a transdisciplinary, participatory and iterative process where practitioners and academics were invited to develop, refine and validate the principles. The process resulted in 10 principles: 1) Ensure active engagement and participation among key stakeholders; 2) Understand the situation (starting points and objectives); 3) Align the intervention with existing organizational objectives; 4) Explicate the program logic; 5) Prioritize intervention activities based on effort-gain balance; 6) Work with existing practices, processes, and mindsets; 7) Iteratively observe, reflect, and adapt; 8) Develop organizational learning capabilities; 9) Evaluate the interaction between intervention, process, and context; and 10) Transfer knowledge beyond the specific organization. The principles suggest how the design, implementation, and evaluation of organizational interventions can be researched in a way that maximizes both practical and scientific impact.

## Introduction

Interventions in the workplace can target individuals, groups or whole organizations, and aim to improve individual, group and/or organizational outcomes by mitigating or preventing problems, or by promoting positive outcomes. Often, these types of interventions aim to achieve the intended outcomes by changing the way work is organized, designed, or managed. These are referred to as “organizational interventions” (Nielsen, 2013). Organizational interventions typically consist of multiple components, sometimes at multiple levels (i.e., employee, group, leader, and organizational; Nielsen et al., 2017), and are typically embedded in their context of application (Montano et al., 2014; Nielsen & Abildgaard, 2013). Examples include job redesign interventions (e.g., Holman & Axtell, 2016), Business Continuity Management aiming to support post-disaster recovery in organizations (Malinen et al., 2019), and participatory occupational health interventions, often including both leader and employee activities (e.g., Framke & Sørensen, 2015).

The fact that organizational interventions involve changing the way work is organized, designed, or managed means that organizational interventions cannot be researched without substantial collaboration between researchers and the organization and its stakeholders (e.g., managers and employees) (Kristensen, 2005). They need to benefit both the organization and the researcher and meet the dual objectives of both parties (Kristensen, 2005). These objectives may differ and follow different logics, even among organizational key stakeholders. They may also be contradictory. Traditionally, the objectives for an intervention researcher in work and organizational psychology have been to evaluate the effects of an intervention (often focusing on *if* something works) and to test theories. The emphasis is on internal validity and the ability to draw causal inferences. The underlying logic dictates that interventions are designed beforehand, preferably based on theory (Fishbein & Yzer, 2003), and then implemented as designed (freezing the interventions). Following this logic, the influence of contextual factors is considered noise that should be minimized (Nielsen, 2017; Nielsen & Miraglia, 2017). Impact on practice is often only considered after the research has been completed.

The main purpose of an organization, however, is not to serve as an arena for researchers, but to produce goods or services (Kristensen, 2005). This does not mean that organizational stakeholders do not see the value of research, but if and when the research process collides with organizational needs, organizational needs will take precedence. For example, an organization may not be willing to wait years to know if an intervention was successful or not, and they may not see the point of “freezing” an intervention if changing it would make it easier to use and/or increase its effectiveness (e.g., von Thiele Schwarz et al., 2016). Thus, even when researchers and organizational stakeholders understand and share each other’s objectives, the logics underlying these ambitions likely differ. This means that research on organizational interventions would benefit from novel approaches that reconcile these apparent contradictory objectives by simultaneously considering both scientific rigour and practical impact.

Such a reconciliation puts specific demands on how the research is conducted; it has considerable impact on the entire organizational intervention process, from design and implementation to evaluation. This discussion paper sets out to address the lack of guidance available for researchers committed to this endeavour. The purpose of this paper is to offer a set of principles, grounded in knowledge from various disciplines, that can guide researchers in designing, implementing, and evaluating organizational interventions that are both scientifically rigorous and practically relevant. In this, the intention is to advance, rather than conclude, the discussion on how to optimize the impact of research on organizational interventions.

The principles contribute to work and organizational psychology in five ways. First, the principles are specifically designed to face the dual and sometimes contradictory objectives of organizational interventions. Traditional guidance for organizational interventions primarily focuses on addressing the concerns of researchers. Less attention is paid to how interventions can directly benefit the organization, or more broadly, how the results can and will be used down-stream (Griffiths, 1999; Rogers, 2008). The principles do *not* suggest that researchers’ concerns for rigour should be abandoned. Instead, the principles suggest striving for rigour in the light of dual objectives, and how real-world impact of organizational research can be managed upstream, that is, as part of the knowledge generation process rather than as a separate process after the research has been conducted. Thus, the principles address the tension between *trustworthiness* and *usefulness* of research evidence.

Secondly, the principles add to the limited understanding of the sustainability of organizational interventions (Kristensen, 2005; Lennox et al., 2018). From an organizational perspective, sustainability is practically inseparable from the real-world impact of an intervention (von Thiele Schwarz et al., 2016). Specifically, the principles highlight how sustainability can be approached throughout the design, implementation, and evaluation of the intervention rather than only once the project is finished.

Thirdly, the principles address all stages of interventions: from design and implementation to evaluation. In this respect, the principles add to the current literature because the existing frameworks that have been developed specifically for organizational interventions have primarily focused on evaluation (e.g., Bauer & Jenny, 2012; Biron & Karanika-Murray, 2013; Nielsen & Abildgaard, 2013; Nielsen & Randall, 2013; von Thiele Schwarz et al., 2016).

Fourthly, the principles take into consideration that organizational interventions are complex, dynamic, and recursive, and consist of multiple components, sometimes at multiple levels (i.e., employee, group, leader, and organizational) and are typically imbedded in a system (the organization) that is also complex in that it includes multiple factors interacting in unpredictable ways (Schelvis et al., 2015). Thus, the principles add to the current limited understanding of how the specific conditions in which organizational interventions operate affect their design, implementation, and evaluation (Griffiths, 1999; Kompier & Aust, 2016; Kristensen, 2005; Nielsen & Miraglia, 2017; Van der Klink et al., 2001; von Thiele Schwarz et al., 2016).

Finally, the principles contribute to work and organizational psychology by synthesizing a breadth of knowledge about organizational interventions that exist in neighbouring fields, including change management, work and organizational psychology, improvement science, implementation science, operations management, occupational health, and applied ergonomics. Therefore, rather than inventing approaches specifically for work and organizational psychology, we build on established knowledge from related fields facing similar challenges, and draw upon different epistemological and ontological points of departure, from positivism to interpretivism and pragmatism.

## Method

Inspired by Mode 2 knowledge production (Gibbons et al., 1994), we brought together transdisciplinary practitioners and academics with experience of organizational interventions and took them through a process to identify key principles for designing, implementing, and evaluating organizational interventions. The core group consisted of 11 academic experts (the authors) from change management, work and organizational psychology, improvement science, implementation science, operations management, organizational theory, occupational health, and applied ergonomics. The researchers were recruited through purposeful snowball sampling of researchers involved in organizational intervention research (Vogt & Johnson, 2011).

Mode 2 knowledge production differs from traditional academic knowledge production (i.e., Mode 1) along five dimensions (MacLean et al., 2002). Firstly, *transdisciplinarity*: While many disciplines research organizational interventions, no one has the definite answer “how to”. We strived to bring together a range of perspectives rather than relying on an in-depth inquiry of knowledge from a single discipline. Secondly, *context of application*: We included practitioners iteratively throughout the process to ensure that the principles reflected real-life issues concerning organizational interventions and to minimize the knowledge generation-knowledge use gap by including knowledge users’ skills and understanding in the knowledge production. Thus, intended users of the knowledge produced are part of a knowledge co-production process rather than mere recipients of the finished product. Thirdly, *heterogeneity and organizational diversity*: Due to the complex nature of organizational interventions, we included practitioners and researchers with experience from various types of institutions and organizations with different approaches to knowledge, ensuring interaction across settings to offer different perspectives on interventions and how knowledge is generated and applied. Fourthly, *reflexivity and social accountability*: Mode 2 knowledge production builds on iterative and reflexive production of knowledge where the potential impact and value (external validity) is integrated in the entire process. Specifically, we used a workshop set-up where we engaged in discussions to make the different perspectives on organizational interventions apparent and transparent. The principles were rigorously questioned and evolved through discussions that allowed participants to reflect on their perspectives in contrast to other disciplines. Finally, *diverse range of quality controls*: We engaged in open discussions of each principle and how to apply them in different practical cases, presented by both core members of the group and invited academics and practitioners. We also included quality controls by presenting the principles at conferences to invite practitioners and academics external to the workshop process to validate the principles.

Following the Mode 2 knowledge production principles, a participatory and iterative approach was used to develop (phase 1) and validate and refine the principles (phase 2). The procedure for the development of the principles is outlined in Table 1, detailing each activity, its purpose, the range of participants involved, as well as the outcome of each step.

**Table 1.** Outline of the procedure for the development of the principles

Purpose	Activity	Participants	Outcome
Phase 1 Development of principles			
Exploration to identify best practices (April 2016)	Workshop with Open Space Technologies^1^ to amend best practices to principles through interactive discussions that examined general applicability, interconnectivity, nomenclature, and perceived importance.	11 researchers across fields (the authors)	15 preliminary principles that summarized the most essential approaches for succeeding with organizational interventions
Substantiation and clarification (April-June 2016)	Working in pairs on a shared platform, the content of the principles was clarified. Each principle was reviewed by the rest of the group members.	The authors	Substantiation and validation of the 15 preliminary principles from each represented research field
Critical revisions (June 2016) Subsequent revisions of principles (July 2016-March 2017)	Work meeting to eliminate overlap and redundancy. Working individually on a shared platform; the content of the principles was clarified.	3 of the authors The authors	15 principles were reduced to 10
Phase 2 External validation and refinement of principles			
External validation for both practical and scientific relevance (April 2017)	One-day workshop using fishbowl methodology^2^ to revise the 10 principles.	The authors and 9 invited practitioners and senior academics	Deeper understanding of ambiguities related to nomenclature and epistemologies; resulted in a final articulation.
Refinement	One-day workshop, iteratively working with the whole group, in pairs, and individually to revise the principles.	The authors	
Further validation with scientific scholars (May and June 2017)	Two symposiums (European Association of Work and Organizational Psychology and Work, Stress and Health; APA-NIOSH). Principles and exemplifying empirical cases were presented. Participants documented and discussed the presence or absence of principles and their feasibility.	5 of the authors and 2 other researchers presented cases and invited feedback from symposium participants (n_total_ = around 100)	Insight into alignment between participants’ perceptions of principles needed for successful and organizational interventions and the principles
Subsequent refinement of principles (June-October 2017)	Refinement based on input from practitioners and academics external to the core group.	The authors	Succinct description of the 10 principles
Further validation with practitioners and researchers (October 2017)	Workshop with practitioners that presented intervention tools that matched the principles.	The authors and 7 invited practitioners and senior academics	Check of principles’ robustness. Final version of principles.

In Phase 1, a two-day workshop was held in the Swedish town of Sigtuna, a key trading and meeting point on the Baltic at the time of the Vikings and hence the name of the principles. Starting from the participants’ current understanding (e.g., Nielsen & Abildgaard, 2013; Nielsen & Randall, 2013; Reed, Howe, Doyle & Bell, 2018; von Thiele Schwarz et al., 2016), a broad range of best practices were identified and explored through reflexive conversations inspired by the Open Space Technology (OST) (Owen, 2008). OST is a participant-driven, real-time approach that relies on self-organization to explore topics of interest to participants. We used this approach to allow participants to move freely in and out of smaller groups, gathering around emerging principles visualized on flipchart papers. Discussions were captured by developing each flipchart. At this stage, the number of principles were allowed to expand and retract, combining old and adding new flipcharts as needed. The flipcharts were then examined by the whole group and through discussions of similarities and differences, they were condensed into a first set of 15 principles. These were further amended and condensed over the following year (see Table 1). In Phase 2, the principles were refined and validated with external experts, including both academics and practitioners, through a series of meetings and workshops (e.g., a symposium at the EAWOP 2017 Conference). Written and oral feedback revealed an overall agreement on the relevance and importance of the principles, but that some were ambiguous. We therefore refined the principles during the following five months, with an additional workshop in October 2017, to finalize the principles.

## The principles

**Figure 1.**
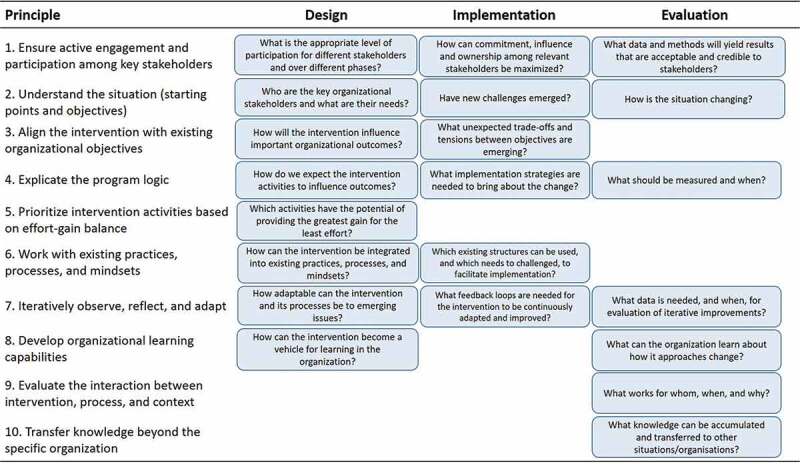
Ten principles for how to design, implement, and evaluate organizational interventions

Organizational interventions often consist of three phases: 1) design, 2) implementation, and 3) evaluation (Tafvelin et al., 2019). The principles cut across the three phases, as illustrated in Figure 1.

***Principle 1: Ensure active participation and engagement among key stakeholders***

This principle recognizes that employees and organizations are not passive recipients of organizational interventions (Nielsen, 2013). They need to shape, manage, and own interventions. Participatory approaches are currently recommended by national and international policy bodies for managing psychosocial risk and for organizational interventions (Nielsen, 2017). Participation is relevant to consider across the design, implementation and evaluation of interventions, and among employees as well as managers at all levels of the organization. The latter includes ensuring senior management support and ownership over the intervention at the appropriate level of the organization (Hasson et al., 2014).

In the design phase, participation can increase the appropriateness of the intervention by ensuring that participants’ expertise is considered in the development of the intervention, e.g., what changes are feasible and appropriate in their workplace (Storkholm, Savage et al., 2019). During implementation, participants are more like to be committed to the intervention if they have had a chance to influence it (Rosskam, 2009). Participation can also facilitate integration into existing work practices and procedures (principle 6) (Tsutsumi et al., 2009). For evaluation, participation increases the likelihood that stakeholders will accept the validity of any findings the evaluation will yield, and commitment to act on them (i.e., evaluability) (Leviton et al., 2010).

What is meant by participation varies greatly, both in terms of the *degree* of influence and in terms of what the participants gain influence *over* (i.e., the content, the process, or the goal of the intervention) (Abildgaard et al., 2018). Based on the substantive evidence supporting active engagement, our proposition is for active forms of participation where researchers and organizational stakeholders, including employees, work closely together throughout the design, implementation, and evaluation of the intervention, enabling influence over all aspects of the intervention, including as co-creative partners (Brydon-Miller et al., 2003; Storkholm, Mazzocato et al., 2019).

Although this principle acknowledges the value of close collaboration and power-sharing (Brydon-Miller et al., 2003), it also acknowledges that the appropriate level of participation varies. For example, the optimal level of participation will vary with the characteristics of the intervention (e.g., the aim), and with contextual factors. These may include cultural differences affecting expectations on degree of participation. For example, participation will be less challenging if it does not deviate from the cultural norms, such as in the European Nordic countries, where there is a long-standing tradition emphasizing collaboration and participation between employer and employees (Gustavsen, 2011).

Degree of participation will also vary during the course of the change process. Participation will be required from different stakeholders at different time points and for different purposes. For example, senior management involvement may be needed when the overall project is designed to ensure the fulfilment of Principles 2 and 3 (Hasson et al., 2018), whereas employee involvement may be most important when the intervention is being implemented on the local level, giving employees and line-managers space and time to integrate the intervention into their work context. Thus, the proposition here is to find the appropriate level of participation across multiple stakeholders. Appropriate level of participation entails understanding the embedded power structures in the organizations (formal/stable hierarchies and informal/fluctuating structures) because they impact the level of influence that the different stakeholders have on the intervention. Not everyone will feel comfortable to speak up, not everyone’s voice will count (Wåhlin-Jacobsen, 2019), and there will be information asymmetries in what people know in the organization that affects the willingness and opportunity to participate, and thus, who has influence over or benefits most from the intervention. As an outsider, such power structures may be tricky to notice, and it may therefore be better to make the default assumption that power-issues are at play.

***Principle 2: Understand the situation (starting points and objectives)***

As outlined in the introduction, organizational interventions are embedded in the organizational context. Therefore, this principle suggests that researchers acknowledge that organizations are social systems, with their own unique dynamics and histories. We propose that the likelihood of a successful outcome is greater when organizational contexts are actively considered in the design, implementation, and evaluation (Nielsen & Randall, 2013; von Thiele Schwarz et al., 2016). Thus, building on disciplines such as engineering and quality improvement, we argue that researchers need to understand the context and take it into account before finalizing the design and starting to implement an organizational intervention (Edwards & Jensen, 2014). In its most basic form, this principle encourages researchers to refrain from conducting organizational interventions if they have not ensured that the organization needs it. For example, introducing a physical exercise intervention may not be the most appropriate in a context where work overload is the main issue.

Understanding the current situation includes considering the work systems, working conditions, history, challenges, and problems, as well as the implicit and explicit goals and intended outcomes of the intervention (e.g., the ideal end state). Such understanding can be achieved through recurrent conversations and negotiations between stakeholders, as well as through more formal assessments describing the situation (e.g., surveys and risk assessments). Knowledge about the organizational context matters for the design and implementation, as well as the evaluation of organizational interventions. First, it helps identifying or designing the most suitable intervention, by clarifying the direction of the change (from where, to where) (Aarons et al., 2011). Then, clarifying the gap between present and desired states may create an urge for change (i.e., “creative tension”), supporting engagement and participation (Principle 1) (Senge, 2006). An understanding of the context can also enable uncovering organizational factors that can make or break the implementation (barriers and facilitators) (e.g., financial constraints, staff turnover, etc.) so that these can be managed. Finally, the knowledge provides information about the starting point (“baseline”), which is essential for evaluation because it makes it possible to track changes over time (Batalden & Davidoff, 2007).

Different stakeholders may not understand the situation in the same way. We do not suggest that all stakeholders must have a fully shared understanding of the situation (i.e., what the problem, the intervention, and the desired outcome are), although it helps to have a mutual agreement on the need for and purpose of the change (Frykman et al., 2014; Storkholm et al., 2017) (i.e., shared sense-making) (Weick, 1995). It is, however, important to understand that there are different perspectives. Research on perceptual distance has shown that a lack of congruence, for example, between managers and employees, has an independent, negative effect on intervention outcomes (e.g., Hasson et al., 2016; Tafvelin et al., 2019). Thus, even if stakeholders do not have a shared understanding of the situation, it is essential that they know if that is the case, so that any misunderstandings can be managed upfront.

***Principle 3: Align the intervention with existing organizational objectives***

As described in the introduction, organizations are not neutral settings for testing research hypotheses, and therefore organizational interventions need to benefit the organization as well as researchers. This requirement for dual benefit means they need to be designed and implemented with consideration of how they contribute to organizational objectives as well as the researchers’ objectives. Alignment with the organization’s objectives serves several purposes. First, alignment helps create engagement by demonstrating how the intervention can contribute to important organizational outcomes. It can reduce the risk of contradictions that emerge when the aim of an intervention is not in line with other objectives (Ipsen et al., 2015; Ipsen & Jensen, 2012). Secondly, it reduces the risk of unintended side effects that emerge when an intervention is designed, implemented, and evaluated without consideration of how it may affect other areas (Bamberger et al., 2016) (e.g., when an intervention benefits one employee group at the expense of another). Finally, aligning objectives is essential to minimize the risk of the intervention becoming a time-limited ancillary project, discontinued once the researchers or a key change agent in the organization move on. Thus, this principle is central for the sustainability of organizational interventions.

Striving for alignment also involves trade-offs and challenges. First, for researchers, it may mean adjusting their research agenda to ensure it is benefitting the organization – or refraining from doing the research in a particular organization where there is no alignment. With regard to the different organizational stakeholders, aligning the intervention with organizational objectives means that the intervention is placed in the landscape of (perceived or real) contradictory and competing organizational objectives, such as those between safety and production (von Thiele Schwarz & Hasson, 2013). This may amplify tensions between stakeholder-groups which, in turn, may pose a barrier to the implementation of the intervention. It may also become an ethical dilemma when researchers and change agents need to favour one organizational objective over another.

Aligning the intervention with organizational objectives does not suggest that the objectives of the intervention automatically change, for example, from focusing on employee health and well-being to focusing on efficiency. Instead, it suggests that discussions about how an intervention might affect various organizational objectives should be considered during the design of the intervention and continually revisited to avoid unexpected agendas derailing the intervention at a later stage. Thus, at a minimum, this principle points to the need to disclose any competing objectives so that they can be managed or monitored to avoid derailment and unsustainable improvements.

***Principle 4: Explicate the program logic***

Given that organizational interventions are dependent on their context, it is essential for the design, implementation, and evaluation to explicate how they are supposed to work. This involves outlining the logical links between the intervention activities and immediate, short-, and long-term outcomes (e.g., Pawson, 2013; Rogers, 2008) including identifying multiple possible intervention activities as well as multiple pathways (Abildgaard et al., 2019). Drawing on the field of program evaluation, this principle suggests explicating a program logic (also known as a program theory, logic model, impact pathway, or theory of intervention) as a way to clarify the proposed theoretical mechanisms that explain why certain activities are expected to produce certain outcomes (Pawson & Tilley, 1997). Program logic focuses on the theory of change, i.e., the *how* and *why* intervention activities may work (Havermans et al., 2016; Kristensen, 2005; Nielsen & Miraglia, 2017), rather than, for example, theories of health (i.e., the relationship between exposure to work factors and employee health).

Program logic is used in the design as well as the implementation and evaluation of an intervention. First, it identifies which intervention activities are most likely to close the gap between the current and desired state. An important part of this is utilizing best available knowledge. Secondly, it guides implementation by clarifying the mechanisms, thereby explicating the implementation strategies needed to support behavioural change. Finally, it is a blueprint for the evaluation, as it describes when and what to measure.

To explicate the program logic, multiple sources of information are needed, so it may benefit from co-creation with stakeholders (von Thiele Schwarz et al., 2018). The development process may differ depending on the extent to which intervention activities are predefined, such as when the change involves implementation of guidelines or an evidence-based intervention. When intervention activities are predefined, they become the starting point for logically identifying appropriate proximal and distal outcomes. When intervention activities are not predefined, the program logic becomes an important part of identifying the intervention activities. This is done by starting from the outcomes and working backwards so that activities that could lead to the desired outcomes are identified (Reed et al., 2014; Saunders et al., 2005). In both cases, the program logic should be considered a hypothesis to be continuously tested and revised throughout implementation.

***Principle 5: Prioritize intervention activities based on effort-gain balance***

Once the program logic has helped to explicate the goals of the intervention and the possible intervention activities, it may be necessary to prioritize between different activities. This principle suggests that the decision of which activities to prioritize should be based on an effort-gain balance analysis. This prioritization involves considering the anticipated impact of an intervention on the one hand and the expected effort needed to realize it on the other (Batalden & Stoltz, 1993; Kotter, 1996; Wilder et al., 2009). Prioritizing activities, therefore, entails striving to strike a balance between the investment (in effort) that an organization is ready to commit to and the potential gains that can be achieved. Understanding this ”return on investment” balance for each intervention activity can inform the prioritization between potential intervention activities and is therefore a calculation integral to the design phase (Cox et al., 2000).

There is a need to identify the potential gains of certain activities in congruence with the goals of the intervention. Potential gains are often evident from the goals of the intervention and the alignment process (Principle 3), or can be illuminated from previous studies. For example, a gain might be improved social support through an intervention involving providing mailmen with mobile phones so they can call each other when on route. Subsequently, the efforts needed to achieve these gains needs to be considered. Efforts include the resources (time, money, emotional and cognitive efforts) involved in bringing about the changes and mitigating the barriers to the design and implementation, e.g., it is not only the financial costs of buying mobile phones, but also ensuring that mailmen have each other’s phone numbers and have the skills to use the mobile phones (i.e., implementation efforts).

Prioritizing and conducting effort-gain analyses is not straightforward. Limited prior experience with implementation or the lack of an organizational learning culture will require additional effort (Kaplan et al., 2011). The advantage of effort-gain balance analyses is that it helps prioritize some activities (low effort-high gain) over others (high effort-low gain). Activities that are low effort-low gain may, however, at times be a feasible starting point, from a motivational perspective, because they can build momentum by showing immediate, albeit limited, results (Cox et al., 2002). High effort-high gain activities may be prioritized when they offer a solution to a central problem, as well as when there is a relative match between the level of complexity of the problem and the solution (Storkholm et al., 2019). The prioritization may also be postponed for implementation later, when the organizational members have further developed their capability to manage change. Overall, using the knowledge of various stakeholders (Principle 1), is vital for ensuring a balanced understanding of efforts-gains.

***Principle 6: Work with existing practices, processes, and mindsets***

During the design, implementation, and evaluation of an organizational intervention, piggybacking on what is already known, already in place, and already done, can help integrate the intervention with the organizational practices, processes, and individual mindsets (Sørensen & Holman, 2014; von Thiele Schwarz et al., 2017). Thus, this principle addresses both organizational (practices and processes) and individual factors. Following this principle involves making the intervention fit with organizational logics and daily work. This fit may reduce the risk of conflict with existing organizational procedures, practices, and mindsets (Storkholm et al., 2017) and facilitate stakeholder engagement (Bauer & Jenny, 2012; Nielsen & Abildgaard, 2013).

This principle is particularly important when planning the implementation because the creation of separate implementation structures is costly, hinders synergies, and prevents the intervention activities from becoming an integrated part of everyday work (von Thiele Schwarz et al., 2015). New structures are easily abandoned once the project is over, which hinders sustainability (Ipsen et al., 2015).

The principle draws on developments in work and organizational psychology (e.g., (Stenfors Hayes et al., 2014; Zwetsloot, 1995)), which in turn build on the integrated management system movement in quality management (Jørgensen et al., 2006; Wilkinson & Dale, 1999, 2002). As an alternative to the conventional praxis of trying to minimize contextual influence, this principle is an example of how the interrelatedness between an intervention and its context should be embraced. For example, if an organization already has a process for working with quality improvement, it may be possible to extend it to include implementation of the intervention activities (von Thiele Schwarz et al., 2017). Other examples of working with existing practices can be to use groups, meetings, and communication pathways that are already in place, rather than creating new practices (Malchaire, 2004).

Nevertheless, it is not always feasible to follow this principle. For example, it is not applicable when the existing processes are part of the problem. That may be the case when the content of the intervention calls for changes of the system, rather than within the system. The implication is that this principle calls for the same careful consideration as when balancing quality improvement, i.e., improvement within a system, and innovation, which challenges the system by breaking new ground (March, 1991; Palm et al., 2014). Thus, it is vital to acknowledge that it may very well be the existing practices, processes, and mindsets that are the root causes of the problems, which in turn makes changing them a core intervention objective. What we are proposing is that the effort involved in breaking new ground such as challenging existing practices, processes, and mindsets should never be underestimated. Thus, challenging them should be done with intention, not by accident.

***Principle 7. Iteratively observe, reflect, and adapt***

Based on the premise that organizational interventions are complex, researchers and organizations need to iteratively observe, reflect, and (frequently) make adaptations to the planned intervention, implementation, or context as the intervention unfolds. This principle calls for ongoing monitoring and evaluation of the intervention progress, as well as the use of that information to improve the intervention content to ensure goal attainment. It also calls for increased attention to factors related to the change process, for example, the frequency of use of problem-solving tools in an intervention, in contrast to only focusing on the intervention’s outcomes.

The principle contrasts with traditional ways of designing, implementing, and evaluating organizational interventions as if they were episodic changes in a static system, with a clearly delineated beginning and end (Nielsen et al., 2010). It builds upon a shift from focusing solely on solving specific problems without questioning the solution (i.e., the intervention) (single-loop learning) to focusing on double-loop learning, which allows the solution, process, and goal to be questioned and modified based on continual monitoring and evaluation (Argyris & Schön, 1996).

The ability of interventions to achieve intended outcomes is mediated by a number of factors related to the interactions between content (intervention activities), process, and context (Pettigrew & Whipp, 1993). Thus, although the program logic (Principle 4) provides a hypothesized model for how it may be played out, this is a hypothesis: How this plays out cannot be fully anticipated beforehand, particularly in interrelated systems where changes in one part of the system can have unintended consequences in another. Therefore, interventions can seldom be fixed and implemented exactly as planned (Chambers et al., 2013). This principle calls for careful attention to what the core components of the intervention are, so that their evolution can be continually monitored, evaluated, and adapted to achieve the intended outcomes. This achievement is, after all, what matters for organizations; they care less about if the intervention is implemented exactly as planned, as long as it works.

Data and analysis are key to ensuring rigour in the process of observing, refining, and adapting an intervention (Storkholm et al., 2019). We suggest iterative cycles in which data concerning the intervention, implementation, context, and outcomes are monitored and used to inform potential adaptations (e.g., Shewhart’s cycle of plan-do-study-act) (Taylor et al., 2014). As a result, organizations and researchers would benefit from a systematic approach to evaluate progress using pragmatic scientific principles (Savage et al., 2018). To ensure this is done rigorously, we suggest to: 1) Use formal and informal methods (surveys, interviews, observations, documents, conversations) to collect data, 2) Mind the time lags as derived from the program logic, 3) Use the information to adapt the design or implementation of the intervention to the context; 4) Conduct small-scale rapid tests of activities, and increase the scale as data accumulate; 5) Identify new systemic challenges that may require the focus of the intervention activities to be revisited; and 6) Consider how intervention activities may adversely affect other parts of the system. This approach ensures a dynamic approach to change. It also positions evaluation as an ongoing process, managed locally by the organization, rather than the domain of the researcher after design and implementation (von Thiele Schwarz et al., 2016; Woodcock et al., 2020).

***Principle 8. Develop organizational learning capabilities***

This principle broadens the scope of researching organizational interventions from a narrow focus on specific study objectives to a broader commitment to facilitate a learning capability within the organization. Building a learning capability ensures that lessons are harvested within the organization to support future change efforts. This includes lessons from designing, implementing, and evaluating an intervention, as well as the tools, infrastructures, and practices developed. Organizational interventions tend to become finite projects which are not sustained over time even though they are often costly (Bernerth et al., 2011). Therefore, researchers involved in organizational interventions need to ensure that individual and organizational benefits are optimized. This principle also highlights the potential added value for organizations collaborating with researchers by ensuring that at least some of the know-how stays in the organization when the researchers leave. For example, it may involve engaging Human Resources staff or internal consultants to deliver intervention components rather than using external experts, or adding components that facilitate transfer of intervention-specific learning to other situations.

This principle is rooted in the disciplines of organizational learning, organizational behaviour, pragmatism, and systems theory, as well as in management concepts such as lean management. Developing a learning capability is essential to an organization’s ability to address future challenges and continually learn from change processes (Nielsen & Abildgaard, 2013). This principle builds on the double-loop learning of Principle 7 and expands it to include triple-loop-learning (i.e., that the organization becomes aware of the processes and structures needed to improve how learning is constructed, captured, disseminated, and incorporated) (McNicholas et al., 2019; Visser, 2007).

***Principle 9: Evaluate the interaction between intervention, process, and context***

If organizational interventions are to be conducted as outlined in the previous principles, it has implications for evaluation, both in terms of evaluation design and analytic approaches. Conceptually, this principle calls for a move away from answering research questions concerning whether an intervention works (isolated from context) to focusing on for whom, when, and why it works, and how it can be improved, building on theories and practice in change management, evaluation science, and organizational science (Pawson, 2013; Pettigrew & Whipp, 1993). By applying this principle, the evaluation sheds light on how a change was brought about: how the intervention interacted with the context (including participants and structures), and how this enabled certain mechanisms to trigger intended outcomes (Pawson, 2013). It contributes to theory building as well as to answering the type of questions practitioners ask.

The evaluation needs to capture the iterative changes to the intervention outlined in Principle 7, as well as the reasons for those changes and the impact they have on outcomes. Yet, in order to meet the objective of contributing both to science and practice, this needs to be done in a way that allows causal inferences as well as accumulation of data across cases. Process evaluation is an important first step in this, addressing research questions such as if employee participation, leadership support or facilitation explains variation in outcomes of an intervention (Biron & Karanika-Murray, 2013). It also calls for research designs beyond pre- and post-measurement, e.g., stepped-wedged designs, propensity scores, and regression discontinuity (Schelvis et al., 2015).

Realist evaluation is another example of how some of the complexities of organizational interventions can be captured. (Pawson & Tilley, 1997). It allows for hypothesized configurations derived from a program logic (Principle 4) to be tested. For example, using multi-group structure equation modelling, one study tested if the impact of a new participatory and structured problem-solving approach (kaizen) on employee wellbeing was explained by whether the kaizen work also included an occupational health perspective and showed that that was indeed the case (von Thiele Schwarz et al., 2017).

There may be a need to move beyond traditional variable-oriented methods and case-studies. One example is statistical process control charts, which build on rigorous time-series analyses to detect if an outcome changes over time over and above the expected natural variation in a pattern that can be attributed to “special-causes” – including, intervention activities (Benneyan et al., 2003; Shewhart, 1930). This analysis allows for testing of research questions. For example, to establish if graphical feedback can have a positive impact on hospital infection trends, or if variation in performance can be reduced by eliminating waste in the work process (Thor et al., 2007).

A third example is configurational comparative methodology (Thiem, 2017). It is a statistical method from the person-/case-oriented family, rather than the variable-oriented approaches that most evaluations of organizational interventions rely on. Coincidence analysis allows assessment of multiple pathways to the same outcome. For example, one study showed that in order to have high intention to change, a positive attitude among staff was always needed, whereas behaviour control was only important under some circumstances (Straatmann et al., 2018). These three examples are very different; yet, they are all examples of evaluation methodologies that combine sensitivity to what works for whom and in which circumstances with scientific rigour (Pawson, 2013; Pawson & Tilley, 1997).

***Principle 10: Transfer knowledge beyond the specific organization***

A cornerstone of organizational research, and what sets it apart from consulting, is the ambition to not only induce change in a single setting but to transfer knowledge from the specific to the general by cumulating learning that can be generalized, abstracted into theory, disseminated, and scaled up. Dissemination and the scaling up of organizational interventions is different from evaluations of interventions that aim to draw generalizable conclusions about the effects of an intervention and where knowledge is accumulated through replication of (the same) intervention. Accumulation through replication requires interventions to be fixed over time and isolated from the context of application. When organizational interventions are approached as outlined in these principles, accumulation through replication is not feasible because the intervention is integrated into, and interacts with, specific organizational contexts and changes over time through ongoing adaptations. This principle builds on the assumption that interventions seldom have an effect independent of the context in which they are used (Semmer, 2006).

In these cases, scalability cannot focus on statistical generalization and accumulation of knowledge through the identification of specific interventions independent of context. Knowledge need to be developed in other ways. This principle outlines that generalization, dissemination, and scalability should rely on analytical generalization, drawing from case study research (Flyvbjerg, 2006; Yin, 2013). This includes addressing research questions such as “What is required to reproduce the impact of the intervention in different settings?” Therefore, we encourage striving for accumulating knowledge, that is, the gradual refinement of phenomena by focusing on the aspects included in the principles, including how various factors interact to produce an outcome, and comparing and contrasting these across studies (Pawson & Tilley, 1997). This accumulation can, for example, be done using methodologies for literature reviews such as qualitative metasyntheses (Docherty & Emden, 1997; Nielsen & Miraglia, 2017). Thus, rather than striving for generalizability, this principle suggests striving for specificity, a gradual increase in the precision of the knowledge of what works for whom and when (Pawson, 2013).

## Discussion

This article set out to propose principles for how to design, implement, and evaluate organizational interventions based on expertise from multiple disciplines, offering suggestions for how organizational interventions can be researched in a way that makes the end result both scientifically rigorous and practically applicable. We propose a way to move the field of organizational interventions forward. Using a Mode 2 knowledge production approach, we draw on our expertise and the literature from multiple disciplines, to propose principles for further empirical testing and development.

In this paper, the principles are presented as discrete entities and in a linear fashion. This is a necessary simplification of a complex process for presentational purposes. The principles may overlap, their order is not self-evident, and they are interrelated. Further research is needed into the contribution of each individual principle, their timing, and the interrelatedness between principles; we hope this paper sparks an interest to advance this agenda.

Viewed one-by-one, the principles are not unique. They reflect the available evidence and/or best practice in one or more research disciplines that are concerned with changes in organizations. Some of them, for example, Principle 1 (Ensure active participation and engagement among key stakeholders), rest on substantial empirical evidence and are common across many disciplines. Others, like Principle 4 (Explicate the program logic), represent established methodological practices in some disciplines (e.g., evaluation science), but not many others. Due to the origins of the principles from various disciplinary backgrounds, the principles are reflected in existing discipline-specific frameworks. For example, Engagement of various stakeholders (Principle 1) and Understanding the situation (e.g., conducting a needs assessment) (Principle 2) and Develop program logic models (Principle 4) are part of the Centres for Disease Control (CDC) Framework for Program Evaluation in Public Health (Centers for Disease Control and Prevention, 1999). However, the CDC framework does not reflect the ambition to meet both research- and organizational objectives, or the dynamic characteristics of organizational interventions. Another example is Brown and Gerhardt’s integrative practice model (Brown & Gerhardt, 2002). The model focuses on the design and formative evaluation of training programs and it emphasizes the need for alignment both with strategy (Principle 3) and work procedures (Principle 6) and iterative development (Principle 7) of training material. Yet, it does not discuss principles such as the use of program logics, choosing activities based on effort-gain balance, or going beyond the training of a specific skill to developing learning capabilities (Brown & Gerhardt, 2002).

Thus, instead of claiming that each principle is unique on its on, we argue that the contribution lies in the convergence of principles across multiple disciplines, and in how they represent a common understanding across a group of experts from various disciplines and research fields, despite their differences in theoretical, empirical, and epistemological backgrounds. Thus, the Sigtuna Principles represent common denominators for researching improvements in organizations that go beyond specific disciplines and may be one step towards a more general theory of organizational interventions.

Do all the principles need to be followed for organizational interventions to be successful? This is an empirical question that calls for further exploration. Our expectation is that the more principles are followed, the better. The degree to which it is feasible to do so is likely to differ between occasions and studies. For example, sometimes, the intervention is predefined, as when guidelines or new legislation is to be implemented, meaning that some principles are not applicable. The number of principles employed will also depend on the mandate that the researcher has in the organization. Sometimes the mandate is restricted to parts of the process, such as when the researcher is only involved in the design or the evaluation phases. This restriction, too, will affect which principles are applicable.

Nevertheless, when combined, these principles offer the potential for a transformative change in the way research into organizational interventions is conducted in at least two interrelated ways. First, it changes the role of researchers and the relationships between the researchers and the organization towards a partnership between equals, where both perspectives are needed to negotiate the inherent contradictions between the objectives. For researchers, adopting a dual perspective implies a change from considering the intervention in isolation, mainly judging the content based on theory or what would yield the highest effect sizes, to thinking about how the practical impact of the change can be maximized. Such an approach includes considering the intervention in relation to the restraints and possibilities of the context where the intervention is set, and the change process, and to determine which activities would result in the most optimal solution given that context (von Thiele Schwarz et al., 2019).

Secondly, the combination of Principles 1–9 on the one hand and Principle 10 on the other, implies a change in the relationship between science and practice. This change involves moving from a one-way street from evidence to practice, where evidence can first be established and then disseminated, implemented, and have an impact, to a constructivist view on knowledge development, where the science base is gradually refined in the interaction with practice (Greenhalgh & Wieringa, 2011). The principles encourage researchers to consider impact upstream, by asking how value for organizational stakeholders can be optimized throughout the design, implementation, and evaluation of the intervention, not just after the research is finished.

The change inherent in applying the principles is not easy, but neither is researching organizations without such considerations: there are whole books dedicated to all the pitfalls involved (e.g., Karanika-Murray & Biron, 2015a). The reasons for derailment include factors related to the intervention itself (e.g., incorrect content), the context (e.g., concurrent organizational changes), and the process (e.g., conflicts and power struggles) (Karanika-Murray & Biron, 2015b), all well-known to organizational researchers. These principles do not solve all of these challenges, but encourage researchers to build relationships with organizational stakeholders so they can be involved in trouble-shooting and problem-solving issues that might threaten to derail the change process – and the research.

The target group for this paper is researchers, yet it is not limited to this group. The principles encourage a way of working in partnership between research and practice, and therefore, the principles are relevant for practitioners, too. In fact, the principles may be of value to practitioners whether a researcher is involved or not. All but the last few principles are related to issues that are of common interest to both practitioners and researchers. The principles are also potentially applicable to other fields, given their development as part of an aspiration to find synergies across communities of practice in various research fields.

The development of the principles followed a structured process focused on harvesting learning from experts from various fields, which increases the trustworthiness of the result. However, they were developed by a relatively small group of people, and although many research fields were represented, not all fields relevant to organizational interventions were. There is still a risk that the principles do not reflect a broader understanding of the phenomena. A thorough validation process with other researchers and practitioners was employed to mitigate this risk.

## Conclusions

This paper presents ten principles that could contribute to the transformation of how organizational interventions are researched, and thereby increase the potential real-world impact. We hope these principles spark interest in the entire intervention process, from the design and implementation to evaluation, and towards a mutually beneficial relationship between the need for robust research and the flexibility needed to achieve change in practice.

## Notes

1. Owen (2008).

2. Priles (1993).
